# Effects of Midazolam and Dexmedetomidine on Cognitive Dysfunction Following Open‐Heart Surgery: A Comprehensive Review

**DOI:** 10.1002/brb3.70421

**Published:** 2025-04-09

**Authors:** Masoomeh Tabari, Ali Moradi, Golbon Asalforoush Rezaieh, Malihe Aghasizadeh

**Affiliations:** ^1^ Department of Anesthesiology, Faculty of Medicine Mashhad University of Medical Sciences Mashhad Iran; ^2^ Clinical Research Development Unit, Ghaem Hospital Mashhad University of Medical Sciences Mashhad Iran; ^3^ Orthopedic Research Center Mashhad University of Medical Sciences Mashhad Iran; ^4^ Vascular and Endovascular Surgery Research Center Mashhad University of Medical Sciences Mashhad Iran

**Keywords:** cognitive dysfunction, dexmedetomidine, midazolam, neuroprotection, open‐heart surgery

## Abstract

**Purpose:**

Patients undergoing open‐heart surgery often face significant challenges in postoperative cognitive dysfunction (POCD). There has been growing interest in understanding how anesthesia medications, such as dexmedetomidine (DEX) and midazolam, impact cognitive function in these patients.

**Method:**

This comprehensive review aims to detail the effect of DEX and midazolam on cognitive outcomes following open‐heart surgery.

**Findings:**

Midazolam, a highly selective and commonly used benzodiazepine for preoperative anxiolytics and sedation has been associated with POCD. However, evidence regarding its impact on cognitive function is vague; some studies suggest a potential link between midazolam administration and cognitive impairment, while others report no effect or even an improvement in cognitive abilities. DEX is a potential neuroprotective agent in cardiac surgery. The effects of DEX on cognitive function, including a reduction in POCD incidence and severity, have been reported in several studies. It modulates the inflammatory responses, attenuates oxidative stress, and preserves cerebral perfusion. Although DEX and midazolam show promising results, their effects on cognitive function following open‐heart surgery are yet to be elucidated.

**Conclusion:**

Various factors, including patient characteristics, perioperative management, and surgical procedures, may influence these outcomes, highlighting the need for further research to better understand the roles of these agents in cognitive function following open‐heart surgery.

## Graphical abstract

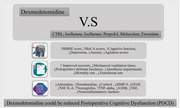

## 1 Introduction

Although open‐heart surgeries save many lives in various cardiac conditions, they are associated with many postoperative psychophysiological risks (Relander et al. 2020). Among the several challenges associated with cardiac surgery, cognitive dysfunction adversely impacts the patients' recovery and quality of life after surgery (Kapoor 2020). Cognitive dysfunction refers to a range of cognitive impairments including a deficit in memory, speed of movement, attention, and executive function (de la Varga‐Martínez et al. 2023).

Postoperative cognitive dysfunction (POCD) is a complex condition primarily affecting older adults after surgery, marked by declines in cognitive function, such as memory, attention, and executive function (Hanning 2005). Symptoms can last from days to months and vary based on surgery type, patient age, preexisting cognitive status, and other risk factors, summarized in Table 1. Acute POCD often manifests in the first few days of postsurgery, sometimes resolving without any intervention, but can lead to chronic forms that significantly impact the quality of life. Chronic POCD is more common in older individuals with preexisting cognitive impairments or comorbidities and may increase the risk of further cognitive decline or dementia. The severity of POCD is assessed through various neuropsychological tests (Blichfeldt‐Eckhardt 2018; Arefayne et al. 2023).

**TABLE 1:** The main risk factor for POCD preoperative, intraoperative, and postoperative.

Preoperative	Intraoperative	Postoperative
Older age	Duration of surgery	Length of stay
Preexisting cognitive dysfunction	Type of anesthesia	Pain management
Comorbidities	Blood loss	Delirium
Low educational level	Surgical stress response	Sleep disturbances
Functional dependence	Intraoperative complications	Mental health issues
Use of certain meds	hypoxia	Poor nutrition
History of stroke	Level of surgical complexity	Social support

Cardiothoracic surgery patients with POCD have a substantial rate of cognitive deficits that persist beyond the immediate postoperative period. The etiology of POCD is multifactorial and may involve a complex interplay between the patient's preexisting characteristics, perioperative factors, and postsurgical complications (Ringle and Wernovsky 2016; Travica et al. 2023; Sabolová et al. 2024). Several risk factors have been identified as potential causes of POCD including the advanced age, preoperative cognitive impairment, intraoperative hypotension, cardiopulmonary bypass (CPB) duration (Bostancı et al. 2024), inflammatory responses (Akar et al. 2023), cerebral microemboli (Zhuang et al. 2023), and microischemic strokes (Perrotti et al. 2019; Fontes et al. 2013). Anesthesia medications in cardiac surgery play a crucial role in managing perioperative complications (Indja et al. 2017) and have been implicated in the development of POCD (Arefayne et al. 2023).

Midazolam, a short‐acting benzodiazepine, is commonly used for preoperative sedation and anxiolysis (Noor et al. 2021). Despite its generally good tolerance, evidence suggests that it may contribute to cognitive impairment (Vu and Smith 2022). Some studies have reported an increased risk of POCD in cardiac surgery patients, while others have shown minimal or even positive effects (Varpaei et al. 2024). The mechanisms underlying midazolam cognitive effects are yet to be elucidated; however, changes in synaptic plasticity (Puig‐Bosch et al. 2022), neurotransmitter systems (Wang et al. 2020), and neuroinflammatory processes (Dominguini et al. 2021) may be involved.

Dexmedetomidine (DEX) is a highly selective α2 adrenergic receptor agonist with some sympatholytic sedative and analgesic properties. It is a promising alternative to traditional sedatives in cardiac surgery (Glumac et al. 2019). The effects of DEX are exerted by binding to presynaptic α2‐adrenergic receptors within the locus coeruleus, ultimately causing ineffective norepinephrine release and sedation (Martin et al. 2008). It modulates the inflammatory responses, attenuates oxidative stress, and preserves cerebral perfusion, making it a promising neuroprotective agent (Van Harten et al. 2012). Evidence shows that DEX improves cognitive function after cardiac surgery, reducing the severity and incidence of POCD (Yan et al. 2022; Mu et al. 2013). Despite the multitude of studies performed on the mechanisms underlying the protective effects of DEX on neural structures that preserve cognition, there is still a lot of work to be done (Huang et al. 2020).

The effects of midazolam and DEX on POCD after cardiac surgery are potentially affected by other anesthesia medications, so a comprehensive evaluation and synthesis of existing literature is critical (Fang et al. 2023). The elucidation of the mechanisms underlying medication's cognitive effects will assist clinicians in developing treatment strategies that reduce cognitive impairment and improve patient outcomes during perioperative periods (Rump and Adamzik 2022; Gold and Forryan 2019; Pappa et al. 2017). This review aims to critically evaluate the current evidence regarding the neuroprotective properties and clinical relevance of DEX and midazolam following open‐heart surgery.

## 2 Pathophysiology of POCD

Symptoms of POCD can manifest as cognitive impairments that occur after a surgical procedure, especially cardiac surgery, and can be severe and multifactorial (Bhushan et al. 2021). Understanding the pathophysiology of POCD involves considering the interplay between many contributing factors and mechanisms. There is increasing evidence that neuroinflammation, which involves the activation of inflammatory pathways in the central nervous system (CNS), plays a crucial role in POCD (Safavynia and Goldstein 2019). An imbalance between the defense mechanisms and the production of reactive oxygen species (ROS) plays an important role due to oxidative stress (Li et al. 2022). Mitochondrial dysfunction significantly impacts neuronal function by impairing the ATP production, leading to decreased energy availability. This dysfunction, along with resultant oxidative stress from increased ROS, can cause considerable neuronal injury and death, contributing to cognitive impairment (Grimm et al. 2016). Additionally, postoperative neuroinflammation results from the activation of brain immune cells (microglia), which release proinflammatory cytokines (such as IL‐1 and IL‐6) and neurotoxic substances, further damaging the neurons. This inflammatory response disrupts synaptic plasticity and neuronal communication, exacerbating the deficits in cognitive function following surgery (Safavynia and Goldstein 2019).

An ischemic reperfusion injury, surgical trauma, and/or systemic inflammation within the brain may all contribute to the generation of ROS. During cardiac surgery, mitochondria, which are the sources of ROS, are particularly vulnerable to damage from oxidative stress (Zakkar et al. 2015). In addition to impairing ATP production, mitochondrial dysfunction triggers disruptions in calcium homeostasis and apoptosis, resulting in neuronal death and synaptic dysfunctions (Baev et al. 2022).

Symptoms of POCD are attributed to oxidative damage to neuronal membranes and synaptic proteins, which compromises neural transmission and synaptic plasticity (Liebert et al. 2016). The antioxidant defense mechanism, which includes enzyme‐based antioxidants (such as catalase and superoxide dismutase) and nonenzymatic antioxidants (such as vitamins C and E and glutathione), mitigates the effects of oxidative stress on neurons (Rizwana et al. 2021). However, a surge in ROS production during the postoperative period can overwhelm the antioxidant defenses, resulting in cognitive impairment and oxidative damage. When performing cardiac surgery, cerebral microemboli can occur in the form of small thrombi, plaques of atherosclerosis, or gaseous emboli that cause POCD and cerebral ischemia (Motshabi‐Chakane et al. 2021). CPB potentially results in microemboli caused by surgical manipulations of the heart and aorta, or dislodgement of atheromatous plaques (Ferrante et al. 2023). These microemboli may embolize the cerebral vasculature, resulting in neuronal damage and microinfarctions.

There is a relationship between the number, size, and composition of microemboli and their impact on cognitive function (Doganci et al. 2013). Smaller emboli are less likely to produce clinically obvious strokes, while larger emboli can result in subtle cognitive deficits that are not detectable with conventional imaging. If repeated, microembolic events can also damage neurons and impair cognitive function. It may be possible to reduce POCD by minimizing the cerebral microembolization during cardiac surgery, such as using emboli detection/protection devices, optimizing CPB parameters (perfusion and suction flow rates, venous reservoir volume, blood temperature, and hematocrit), and using surgical techniques carefully (Patel et al. 2016). Hemodynamic instability, which includes fluctuating blood pressure, oxygen delivery, and cerebral perfusion, compromises cerebral autoregulation, predisposing to ischemia and cerebral hypoperfusion (Elsayed and Abdul Wahab 2022). Various factors can affect hemodynamics during cardiac surgery, including surgical manipulations, intravascular volume and cardiac output changes, and vasoactive medication administration (Nogueira et al. 2021).

Cardiac surgery often results in significant hemodynamic disturbances, particularly hypotension during anesthesia induction, CPB initiation, and weaning (Roberts et al. 2023). This hypotension can impair cerebrovascular autoregulation, reducing cerebral blood flow and oxygen delivery, while hypertensive spikes risk intracerebral hemorrhages and swelling. Older patients, who typically have preexisting cerebrovascular conditions and impaired autoregulation, are especially vulnerable (Singh et al. 2023). Prevention of POCD and cerebral hypoperfusion can be achieved through personalized blood pressure targets, temperature regulation, and avoiding excessive fluids during surgery (Lomivorotov et al. 2022).

## 3 Anesthesia Management in Cardiac Surgery

Managing anesthesia during cardiac surgery is one of the most important aspects of perioperative care in order to maximize patient safety, optimize surgical conditions, and minimize the complications (Bolliger et al. 2019). A specialized anesthetic approach is essential for cardiac surgery, including an evaluation of medical history, intraoperative monitoring, and choice of appropriate anesthetic agents (Samy and Taksande 2024). The discussion aims to explore the principles of anesthesia management in cardiac surgery, examine techniques such as preoperative evaluation and intraoperative monitoring, and discuss the role of specific anesthetics. A comprehensive preoperative assessment is critical to identify comorbidities, cardiac function, and optimize the patient's overall health (Kim et al. 2016). Multiple comorbidities such as valvular heart disease, heart failure, and coronary artery disease often impact the anesthetic management of cardiac surgery patients (Mihalj et al. 2020).

A thorough evaluation of cardiac function, including the left ventricular ejection fraction (LVEF), arrhythmias, and valvular pathology, is an essential portion of the perioperative process in determining the perioperative risk stratification and intraoperative hemodynamic management (Lee et al. 2019).

Continuous electrocardiography (ECG) monitoring can early detect myocardial ischemia, abnormal conduction, and arrhythmia patterns (Ware et al. 2021). Real‐time invasive arterial blood and central venous pressure monitoring can help in titrating the vasoactive medications and fluid management. Monitoring the mixed venous oxygen saturation (SvO_2_), right atrial pressure (RAP), and central venous pressure (CVP) is possible through central venous catheterization, providing the preload, volume status, and tissue oxygenation information (Jasińska‐Gniadzik et al. 2022). Trans‐esophageal echocardiogram (TEE) is an excellent tool for intraoperative imaging modality that allows real‐time dynamic assessments of cardiac structure and function (Poddar et al. 2020). It allows early intraoperative decision‐making regarding volume status, valve function, and ventricular function and can detect perioperative complications like valve dysfunction, myocardial ischemia, and air embolism (Mahmood and Shernan 2016). A new monitoring technique, near‐infrared spectroscopy (NIRS), measures the regional oxygen saturation (rSO_2_) of the tissues and provides information about tissue perfusion and cerebral oxygenation. Continuous monitoring of cerebral oxygenation using NIRS helps optimize intraoperative management strategies to maximize cerebral oxygen delivery (Pisano et al. 2020).

Neuroprotection focuses on minimizing the neurological injury during cardiac surgery, especially during periods of reduced cerebral perfusion associated with CPB and other stressors. The Bispectral Index (BIS) utilizes EEG to measure anesthesia depth, helps prevent awareness, and optimizes drug delivery while avoiding hemodynamic fluctuations. There are some advantages of EEG‐based neuromonitoring (Tran et al. 2018). EEG provides rapid detection of changes in brain function and enables immediate intervention. Assessment of cerebral ischemia facilitates early identification of ischemic events to prevent prolonged hypoperfusion and subsequent cognitive decline. By offering continuous insights into brain status, anesthesia providers can adjust their techniques as needed for improved patient outcomes (Paraschiv et al. 2024).

Cardiac surgery balanced anesthetic agents are selected based on the myocardial function, hemodynamic stability, neuroprotection, and postoperative recovery (Perkowski and Oyama 2015). Intravenous administration of anesthesia‐inducing agents such as benzodiazepines, etomidate, and propofol, minimizes hemodynamic effects and promotes rapid induction of anesthesia (Liu et al. 2017). Propofol, a short‐acting intravenous hypnotic agent, is frequently used during cardiac surgery for induction and maintenance of anesthesia due to its favorable hemodynamic profile and neuroprotective qualities (Irwin et al. 2020). Various volatile anesthetics provide cerebral protection, cause cardiac depression, and induce blood vessel dilation when inhaled (Kotani et al. 2008). As inhalational anesthetics provide favorable hemodynamic stability, titratable depths of anesthesia, and rapid recovery. It may be used for patients with unstable hemodynamics or compromised myocardial function undergoing cardiac surgery.

## 4 Role of Midazolam in Anesthesia for Cardiac Surgery

Midazolam has hypnotic, amnestic, sedative, and anxiolytic properties. It may be of value during the perioperative period in patients undergoing cardiac surgery (Athanassoglou et al. 2022). For optimal patient care, it is essential to understand the pharmacology of midazolam, its indications, and its impact on cognitive function following surgery (Azeem et al. 2018). Evidence suggests that midazolam acts as an allosteric modulator of the gamma‐amino‐butyric acid (GABA) receptor type A, thereby potentiating the inhibitory GABAergic neurotransmission across the brain (Newman et al. 2015). Aside from sedation and anxiolysis, midazolam produces muscle relaxation by enhancing GABA effects (Prommer 2020). A significant difference between midazolam and other benzodiazepines is its quick effect and relatively short half‐life because it is rapidly metabolized by hepatic enzymes, primarily cytochrome P450 3A4 (Bond and Lader 2016). The extensive hepatic hydroxylation and conjugation of midazolam leads to the formation of pharmacologically active metabolites, including 1‐hydroxy midazolam and 4‐hydroxy midazolam (Nguyen et al. 2016).

Metabolism of the metabolites leads to inactive glucuronide conjugates excreted through the kidneys. The impairment of the liver can result in a prolonged elimination half‐life of midazolam and its metabolites and, hence, may necessitate dose adjustments (Diep et al. 2017). The use of midazolam for preoperative anxiety management, induction of anesthesia, and maintaining intraoperative sedation is common in cardiac surgery (Du et al. 2019). The amnestic properties of midazolam are especially beneficial for patients experiencing distress or discomfort during invasive procedures (Jeon et al. 2018). Midazolam, as an adjunct to the induction of anesthesia, is often used to facilitate the smooth transition from temporary to permanent anesthesia (Ghazal et al. 2019). As a result of its sedative and anxiolytic properties, midazolam can be used to reduce the stress response during endotracheal intubation, surgery, and laryngoscopy.

Intraoperative administration of midazolam may be intermittent or continuous to prevent hemodynamic instability while maintaining adequate sedation (Das and Ghosh 2015). Postoperatively, midazolam is used for sedation in the intensive care unit (ICU) for agitation and delirium management (Jerath et al. 2017). As a result of its rapid onset and short duration of action, midazolam is suitable for titratable sedation regimens when a critical patient is receiving mechanical ventilation or medical support. Midazolam effect on cognitive function postoperatively, and in particular after cardiac surgery, has been debated and investigated (Patel et al. 2015). As a sedative and amnestic medication, midazolam may help reduce preoperative anxiety and administer intraoperative relaxation, but POCD has been brought up as a concern. The symptoms of POCD include cognitive deficits as well as executive and psychomotor dysfunction that persist long after surgery. Several studies have examined the association between POCD and midazolam administration in cardiac surgery patients (Huang and Zhang 2023; Tang et al. 2019). Table 2 summarizes several studies indicating the link between midazolam and increased risk of POCD (Lertkovit et al. 2022). In a clinical trial involving elective on‐pump CABG candidates, 42 participants were randomly assigned to two groups receiving midazolam or DEX. Both groups underwent preoperative cognitive assessments using the Persian versions of the Mini‐Mental State Examination (MMSE) and Wechsler Memory Scale (WMS). Although presurgery scores showed a homogeneity between the two groups, a significant difference was observed in WMS scores 30 days postsurgery, with the midazolam group exhibiting a greater cognitive impairment compared to the DEX group (87.60 vs. 103.53; *p* = 0.005). However, postsurgery MMSE scores showed no significant difference between the two groups (24.80 vs. 23.55; *p* = 0.394) (Rajaei et al. 2019). Variations in the study design, patient characteristics, surgical techniques, and perioperative management protocols may explain the inconsistent results. Furthermore, the subjective nature and heterogeneity of cognitive assessment tools make it difficult to draw concrete conclusions about the cognitive effects of midazolam. As a result of midazolam sedative effects, it alters neurotransmitter systems, disrupts sleep architecture, and interferes with memory consolidation (Leong et al. 2022).

**TABLE 2:** Clinical studies evaluating the effect of midazolam on cognitive function.

Study	Year	Design	Sample size	Groups	DEX outcomes
Rajaei et al. (2019)	2018	RCT	42	Midazolam (0.05–0.1 mg/kg) vs. DEX (1 µg/kg)	DEX might have a lower impact on cognitive function than might midazolam among patients undergoing CABG
Taipale et al. (2012)	2012	Observational study	122	Midazolam	Logistic regression models revealed that for every additional milligram of midazolam administered, the patients were 7%–8% more likely to develop delirium
Yoshimura et al. (2023)	2023	RCT	16185	Midazolam vs. no midazolam	Intraoperative administration of midazolam may not induce postoperative delirium in patients undergoing cardiac surgery
Erol et al. (2020)	2020	RCT	50	Midazolam vs. no midazolam	The use of midazolam in induction and perfusion in patients undergoing CPB could reduce the development of delirium in the postoperative period

Another study explored the link between preoperative administration of midazolam and the onset of postoperative delirium. Involving 122 elective CABG patients, the analysis found that each additional milligram of midazolam increased the likelihood of developing delirium by 7%–8%. Furthermore, multivariate logistic regression showed that this association was not influenced by established risk factors such as age and peripheral vascular disease (Taipale et al. 2012).

A retrospective observational cohort study in 2023 examined the relationship between the intraoperative administration of midazolam and postoperative delirium in patients undergoing cardiovascular surgery in Japan. Results indicated that patients who received midazolam had a slightly longer ICU stay (3.5 days vs. 3.3 days; *p* < 0.001) and longer overall hospitalization (31.5 days vs. 29.4 days; *p* < 0.001), but experienced a lower incidence of postoperative nausea and vomiting (OR 0.92; 95% CI 0.85–0.99; *p* = 0.03) (Yoshimura et al. 2023).

Another study investigating the preoperative midazolam administration on the incidence of postoperative delirium in patients undergoing open‐heart surgery showed a significantly higher rate of delirium before extubation in patients who did not receive midazolam (*p* = 0.01), suggesting that midazolam use during induction and perfusion in patients undergoing CPB may help reduce the occurrence of delirium in the postoperative period (Erol et al. 2020). The rapid onset and short duration of action of midazolam may result in residual sedation and amnesia in some patients, particularly the elderly or those with impairments in drug metabolism (Taipale et al. 2012; Kapoor 2020). ALthough the cognitive effects of midazolam remain unclear, the judicious use of medication in cardiac surgery is imperative for achieving optimal perioperative outcomes (Hong et al. 2008).

## 5 Role of DEX in Cardiac Surgery

DEX is a highly selective α_2_‐Adrenergic receptors agonist with sedative, analgesic, anti‐anxiolytic, and sympatholytic effects (Zhang et al. 2022). The drug's unique pharmacological profile makes it a valuable perioperative agent in cardiac surgery patients. Understanding the pharmacokinetics, perioperative applications, and neuroprotective properties of DEX following open‐heart surgery is crucial to optimizing patient care (Jin et al. 2020). As an alternative to benzodiazepines and opioids, DEX offers a superb option for perioperative sedation without causing significant respiratory depression (Castillo et al. 2020). Analgesia and sedation are dose‐dependent effects of DEX, producing a more excellent analgesia and a more profound sedation at higher doses. In addition to bradycardia and hypotension, it is necessary to titrate DEX and monitor the patient's hemodynamics while it is administered due to its sympatholytic properties (Chima et al. 2022).

Considering the advantageous pharmacokinetic profile of DEX (rapid action time and short elimination half‐life), it can be used for precisely controlled sedation and quick consciousness regaining after discontinuation (Beckman et al. 2017). DEX is used in many perioperative settings in cardiac surgery, including the preoperative patient sedation, adjunct anesthesia during the operation, and postoperative analgesia and sedation during the recovery period (Zhang et al. 2015). It is administered preoperatively to facilitate patient cooperation, reduce preoperative stress, and provide sedation and anxiolysis (Ji et al. 2013). DEX administration during intraoperative anesthesia may reduce the requirement to use volatile anesthetics, opioids, and other sedative agents in order to decrease the depth of anesthesia (Momeni et al. 2021). It reduces hemodynamic instability during surgery the stress response to anesthesia, and facilitates smooth emergence from anesthesia (Likhvantsev et al. 2021). During the postoperative period, DEX reduces pain and increases sleepiness in the ICU, especially for patients who need hemodynamic support or mechanical ventilation (Mahmoud and Mason 2015). In critically ill patients, DEX can provide sedation without causing respiratory depression, thus facilitating early extubation and maintaining comfort (Wu et al. 2018).

After open‐heart surgery, DEX may also promote neuroprotection and preserve cognitive function by acting as a sedative and analgesic. Studies have shown that DEX can reduce perioperative neurocognitive deficits by inhibiting neuroinflammation, reducing oxidative stress, and preserving cerebral perfusion (Li et al. 2023). In addition to suppressing proinflammatory cytokine production, DEX inhibits microglial activation and modulates astrocyte function (Carr et al. 2018). Inhibition of neuroinflammatory responses by DEX prevents neurodegenerative processes and mitigates the effects of postoperative cognitive impairment (Wang et al. 2023). Following cardiac surgery, oxidative stress, caused by systemic inflammation, surgical trauma, and ischemia‐reperfusion injury, has been found to contribute to cognitive dysfunction and neuronal injury (Tian et al. 2021). With DEX's antioxidant properties, including its ability to scavenge free radicals while also boosting the production of endogenous antioxidant enzymes, the oxidative damage may be mitigated and cognitive function preserved in high‐risk patients (Alam et al. 2017). Additionally, DEX maintains cerebral autoregulation and regulates cerebral perfusion, thus reducing the risk of postoperative cognitive deficits caused by hypoperfusion after surgery (Slupe and Kirsch 2018). DEX may result in improved long‐term neurocognitive function after cardiac surgery when combined with cerebral oxygen delivery optimization and reduction in neurovascular dysfunction (Lin et al. 2019; Berger et al. 2018).

Clinical studies on POCD in the context of cardiac surgery aim to identify its prevalence, risk factors, and underlying mechanisms, which manifest as deficits in cognitive, attentional, and executive functions following the procedure. These studies utilize various neuropsychological evaluation tools, including standardized cognitive tests and subjective patient‐reported outcomes, to assess cognitive performance before, during, and after surgery. Additionally, research has explored the neuroprotective effects of anesthesia medications including DEX (Gong et al. 2018; Pournajafian et al. 2020; Lei et al. 2020; Gao et al. 2021; Xin et al. 2021; Wang et al. 2022), midazolam (Rajaei et al. 2019), propofol (Metry et al. 2019; Pournajafian et al. 2021), and others (Fu et al. 2024) in order to seeking ways to alleviate POCD effects and preserve the cognitive function (shown in Table 3).

**TABLE 3:** Clinical studies evaluating of DEX on cognitive function.

Study	Year	Design	Sample size	Groups	DEX Outcomes
Gong et al. (2018)	2018	RCT	97	DEX vs. CTRL	Increased MMSE score Increased MoCA scores Quicker recovery from anesthesia Shorter mechanical ventilation times Lower adrenaline, noradrenaline, and cortisol levels after surgery
Kang et al. (2018)	2018	RCT	80	DEX + isoflurane vs. isoflurane	No significant difference in cognitive function between the two groups postsurgery. Significantly lower Matrix Metallo Proteinase‐9 (MMP‐9) in DEX group Significantly lower GFAP levels in DEX group
Metry et al. (2019)	2019	RCT	60	DEX vs. propofol	No significant difference in MMSE scores postextubation between the two groups
Rajaei et al. (2019)	2019	RCT	42	DEX vs. Midazolam	Significant reductions in cognitive impairment (lower WMS) 30 days postsurgery No significant difference in MMSE scores
Zaho et al. (2020)	2020		120	DEX vs. Propofol	Significant reduction in postoperative delirium incidence Decreased risk of off‐pump surgery
Pournajafian et al. (2021)	2020	RCT	72	DEX vs. CTRL	DEX infusion before surgery did not prevent POCD in elderly patients undergoing orthopedic surgery
Pournajafian et al. (2020)	2020			DEX vs. CTRL	DEX group displayed lower levels of neuron‐specific enolase (NSE) and IL‐6 on the day following surgery
Lei et al. (2020)	2020		31 study 2077 patient	DEX vs. Propofol	Lower POCD incidence on Day 7 Lower anesthesia requirements
Gao et al. (2021)	2021	RCT	40	DEX vs. CTRL	Significantly lower incidence of POCD at POD7 and POD30 Increased neuroglobin levels Higher MMSE scores.
Xin et al. (2021)	2021		60	DEX vs. CTRL	Significant decrease in the incidence of POCD DEX was associated with a reduction in TNF‐alpha, MMP‐9, and GFAP levels
Li et al. (2021)	2021	Meta‐analysis	14 study 1029 patient		Significantly lower postoperative POCD rates than controls Improved MMSE scores
Norden et al. (2021)	2021	RCT	63	DEX vs. Placebo	Significant reduction in postoperative delirium Lower mortality rate
Wang et al. (2022)	2022	RCT	80	DEX vs. CTRL	significant increase in MMSE and MoCA scores 24 and 72 h after DEX administration Reduced TNF‐α, S100β, NSE, cortisol, norepinephrine, and IL‐6
Singh et al. (2022)	2022		969	DEX vs. propofol	Significantly lower incidence of delirium in DEX group Higher transfusion rate in propofol group No significant differences in ICU and hospital stays No differences in mortality rates No significant differences in midazolam usage
Wang et al. (2024)	2024	Meta‐analysis	12 study 1200 patient		Significant reduction in cognitive dysfunction and postoperative delirium
Fu et al. (2024)	2024		132	DEX + etomidate vs. etomidate	Lower depression, anxiety, and agitation scores on the MMSE Reduction in plasma concentrations of neuroinflammatory markers and NSE

## 6 Clinical Implications and Future Directions

Several clinical studies have demonstrated that cardiac surgery can affect cognitive function, which has important implications for clinical decisions and perioperative care. Patients with POCD often face significant challenges in terms of recovery, functional independence, and overall quality of life after surgery (Hasan et al. 2020). Understanding the factors contributing to POCD, the associated mechanisms, and the potential interventions that can be undertaken to optimize patient care will contribute to improving patient outcomes after cardiac surgery (Lin et al. 2020). Research on POCD has important clinical implications, including identifying modifiable risk factors that contribute to cognitive dysfunction after cardiac surgery. Several factors have been implicated in the pathogenesis of POCD, including perioperative hemodynamic instability, advanced age, inflammation, preexisting cognitive dysfunction, and oxidative stress (Bowyer et al. 2014). Using knowledge, clinicians can develop targeted interventions to mitigate these risk factors and reduce the severity and incidence of POCD (Newman et al. 2014). Neuroprotective agents, anesthesia management optimization, hemodynamic optimization, and improved recovery protocols can help minimize improved outcomes and cognitive impairment in cardiac surgery patients (Muedra et al. 2022). Standard neuropsychological assessment protocols and cognitive screening tools have also been developed to detect and monitor cognitive dysfunction in cardiac surgery patients early (Kotekar et al. 2018). A routine cognitive assessment can assist clinicians in identifying patients at risk of perioperative, perioperative, and postoperative POCD to tailor interventions accordingly (Berger et al. 2018). Furthermore, taking patient‐reported outcomes into account and subjective measures of cognitive function is an effective way to provide patients with valuable insight into their cognitive experience and facilitate customized care tailored to their unique needs (Benesch et al. 2021). There is increasing evidence as to the potential role that neuroprotective agents like DEX may play in preserving cognitive function after cardiac surgery, particularly in the case of successful outcomes following a cardiac procedure (Pisano et al. 2021). DEX works on cerebral hypoperfusion, neuroinflammation, and oxidative stress, decreasing the risk of POCD through its cerebrovascular anti‐inflammatory and antioxidant properties (Abraham 2014). Optimal dosing regimens, the timing of administration, and selection criteria for patients who will benefit from DEX for cardiac surgery are critical to maximizing its neuroprotective effects and improving cognitive outcomes after cardiac surgery. It is also necessary to conduct ongoing clinical trials and prospective studies to determine whether perioperative interventions effectively attenuate or prevent POCD (Hogue et al. 2006). The results of randomized controlled trials (RCTs) evaluating the effects of perioperative care protocols, anesthesia management strategies, and neuroprotective agents on cognitive function will be valuable for clinical practice and informing treatment decisions in clinical settings. Long‐term studies measuring cognitive recovery and functional outcomes in cardiac surgery patients will also assist in identifying cognitive resilience predictors and inform strategies to optimize neurocognitive health after surgery.

## 7 Conclusion

As a result of the comprehensive review of clinical studies, it has become evident that POCD in cardiac surgery patients must be properly understood and addressed during the perioperative process. The previous clinical trials provide patients with POCD with a range of multifactorial symptoms with significant implications for their quality of life and outcomes. Identifying modifiable risk factors, implementing standardized cognitive assessment protocols, and exploring novel interventions may help clinicians reduce the incidence and severity of POCD in cardiac surgery patients and improve their cognitive outcomes. The findings from this study indicate that benzodiazepines, particularly midazolam, are frequently utilized in the preoperative phase for anxiolysis and sedation in relation to POCD. However, using midazolam in older adults where more risk factors are observed and underlying diseases may be present is not recommended. Additionally, DEX, recognized for its neuroprotective properties, may help reduce the incidence and severity of POCD. However, further investigation is necessary to explore the mechanisms underlying cognitive dysfunction associated with midazolam.

## Author Contributions

**Masoomeh Tabari**: conceptualization; supervision. **Ali Moradi**: methodology, investigation, formal analysis. **Golbon Asalforoush Rezaieh**: writing–original draft, project administration. **Malihe Aghasizadeh**: writing–review and editing, formal analysis, investigation.

### Peer Review

The peer review history for this article is available at https://publons.com/publon/10.1002/brb3.70421

## Data Availability

The data that support the findings of this study are available from the corresponding author upon reasonable request.
